# Applying a Deep-Learning-Based Keypoint Detection in Analyzing Surface Nanostructures

**DOI:** 10.3390/molecules28145387

**Published:** 2023-07-13

**Authors:** Shaoxuan Yuan, Zhiwen Zhu, Jiayi Lu, Fengru Zheng, Hao Jiang, Qiang Sun

**Affiliations:** Materials Genome Institute, Shanghai University, Shanghai 200444, China; shaoxuanyuan@shu.edu.cn (S.Y.); zhiwenzhu@shu.edu.cn (Z.Z.); jiayilu@shu.edu.cn (J.L.); fengruzheng@shu.edu.cn (F.Z.); jianghao666@t.shu.edu.cn (H.J.)

**Keywords:** YOLO, keypoint recognition, computer vision, scanning tunneling microscope

## Abstract

Scanning tunneling microscopy (STM) imaging has been routinely applied in studying surface nanostructures owing to its capability of acquiring high-resolution molecule-level images of surface nanostructures. However, the image analysis still heavily relies on manual analysis, which is often laborious and lacks uniform criteria. Recently, machine learning has emerged as a powerful tool in material science research for the automatic analysis and processing of image data. In this paper, we propose a method for analyzing molecular STM images using computer vision techniques. We develop a lightweight deep learning framework based on the YOLO algorithm by labeling molecules with its keypoints. Our framework achieves high efficiency while maintaining accuracy, enabling the recognitions of molecules and further statistical analysis. In addition, the usefulness of this model is exemplified by exploring the length of polyphenylene chains fabricated from on-surface synthesis. We foresee that computer vision methods will be frequently used in analyzing image data in the field of surface chemistry.

## 1. Introduction

The recent developments in on-surface reactions have enabled the fabrications of complex nanostructures down to atomic precision [1,2,3,4,5]. Notably, scanning tunneling microscopy (STM) imaging has proven to be one of the most powerful tools for investigating the on-surface reactions, providing high-resolution, molecule-level insights [4,6,7,8]. It offers the capability of identifying reactants, products, and important intermediates and thus unraveling the underlying reaction mechanisms of the atomic-scale processes occurring on surfaces. STM works by scanning a sharp tip over the surface of a material while applying a bias voltage. By measuring the tunneling current between the tip and the surface, STM can create topographic maps that reveal the atomic and molecular species on the surface. The resulting images offer fertile information about the size, shape, orientation, and distribution of molecules or atomic structures, enabling researchers to study molecular adsorption, self-assembly, and reactions [9,10,11,12,13,14,15,16].

Despite advances in STM, the analysis of STM images still heavily relies on manual inspection and interpretation, which is labor intensive, time consuming, and often subjective [17]. Experienced researchers rely on their expertise to identify individual molecules or atomic structures in the images. This manual approach becomes increasingly challenging as the complexity and size of the datasets grow, limiting the scalability and efficiency of the analysis process. Additionally, the lack of standardized criteria for image analysis inevitably introduces variability and inconsistency in analyzing similar experiments conducted by different researchers, or even in different researchers analyzing the same data.

Recently, machine learning techniques, particularly computer vision, have emerged as promising tools in material science research for automating the analysis and processing of image data [18,19,20,21,22,23,24,25]. Machine learning has achieved remarkable advancements even in the field of medical and biological sciences [26,27,28]. By leveraging deep learning algorithms and their image recognition capabilities, computer vision techniques offer the potential to overcome the limitations of manual analysis and provide accurate results with much improved efficiency and consistency [29,30]. Computer vision techniques applied to STM images involve training deep learning models on labeled datasets to recognize and classify individual molecules or atomic structures [17]. The deep learning models can then be used to automatically identify and analyze properties of interest in STM images. By applying computer vision techniques in the high-resolution STM imaging, we can extract valuable information of nanoscale phenomena, such as molecular size, shape, orientation, or patterns, with significantly enhanced efficiency and accuracy of image analysis.

In this work, we propose a method for analyzing molecular images using computer vision techniques. Our approach aims to automate the analysis of STM images and alleviate the efforts associated with manual inspection by developing a lightweight deep learning framework based on the YOLO (You Only Look Once) algorithm. The YOLO algorithm [31] is a one-stage method that stands for You Only Look Once. It is a neural network that can output results by looking at an image only once. YOLO has released seven versions so far, with YOLOv1 laying the foundation for the entire YOLO series and subsequent YOLO algorithms constantly improving and innovating on it. The YOLO algorithm uses a single CNN model to achieve end-to-end object detection. The core idea is to use the entire image as the input of the network and directly regress the position of bounding boxes and their categories at the output layer. We can accurately label each molecule in the STM images with its keypoints, enabling molecule classification and counting and statistical analysis of molecular properties in an instant manner (less than one second for one image with hundreds of molecules). In addition, the usefulness of this framework is exemplified by its application in exploring the length of polyphenylene chains fabricated from on-surface synthesis.

## 2. Discussion and Results

**Figure 1:**
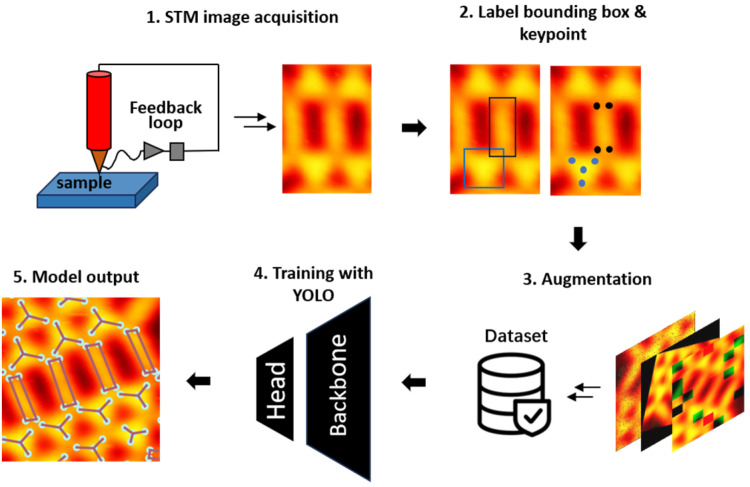
Overall workflow starting from STM image acquisition to the dataset preparation and training with the YOLO model to the model output.

The workflow of our deep learning framework consists of five modules: standard STM image acquisition, bounding box and keypoint labeling, dataset preparation, model training, and model output. The final outputs contain the information of keypoints on each recognized molecule which are used for further statistical analysis. The overall workflow is illustrated in Figure 1.

STM is a powerful technique for imaging molecules on surfaces. To acquire molecular resolved images, we operated STM under ultra-high vacuum (UHV) conditions for minimizing influences such as contaminations. Due to the principle of STM, the obtained images contain abundant molecular and atomic information including molecular electronic properties and the apparent heights of molecules above the surface. Currently, the application of machine learning models in the keypoint recognition of STM molecular images is scarce [32]. Therefore, we harnessed the YOLOv7 model to incorporate the functionality of keypoint recognition.

We opted for YOLOv7 because of its impressive accuracy and superb capability to handle various input images, in addition to its versatility to be deployed on different platforms [33,34]. We have also compared three different deep learning models and confirmed the outstanding performance of YOLO at a reasonably low cost. (See more details of the comparison in Table S3). The architecture of YOLOv7 is composed of four main components: Backbone, Neck, Head, and Loss [35]. The Backbone is primarily responsible for feature extractions and uses an E-ELAN computational block. This block has been designed to constantly improve the network’s learning ability without disrupting the original gradient path. It typically employs CSPDarknet53, which leverages the Cross Stage Partial Network (CSP) structure to enhance efficiency and accuracy in feature extractions. CSPDarknet53 comprises multiple convolutional layers and residual blocks, enabling multi-scale feature extraction. The Neck serves as a feature fusion module that employs both the PANet (Path Aggregation Network) and the BiFPN (Bidirectional Feature Pyramid Network). The PANet combines fine-grained features from lower levels with semantic information from higher levels through up-sampling and down-sampling operations, thereby enhancing the detection performance of small objects and the perception of image details. The BiFPN introduces bidirectional pathways for inter-layer feature interaction, balancing the contributions of different levels through adaptive weight adjustment, which results in improved accuracy and robustness in object detection. The Head component in YOLOv7 includes an auxiliary head in addition to the primary one, which aids in better training and supervision of detection tasks. The Head is tasked with making predictions about object class, position, and keypoints. The Loss function includes classification loss, box regression loss, and object presence loss, all of which are used during the training process. YOLOv7 also implements a compound model scaling approach, where the network width and depth are scaled in coherence for concatenation-based models, facilitating optimization for different sizes and applications. 

While there are various publicly available annotated datasets, there is no molecular dataset specifically designed for training nano-objects of STM imaging. To train our model effectively and achieve good results, we need to require a large amount of labeled data as inputs into the ML models. However, manually annotating thousands of STM images as a dataset is time consuming and impractical. We employed data augmentation techniques after manually labeling a small number of STM images of single molecules. To enrich our dataset, we generated a training dataset by maintaining the labels using data augmentation techniques, resulting in thousands of generated images. More detailed explanations of the data augmentation methods are provided in the supporting Table S1.

**Figure 2:**
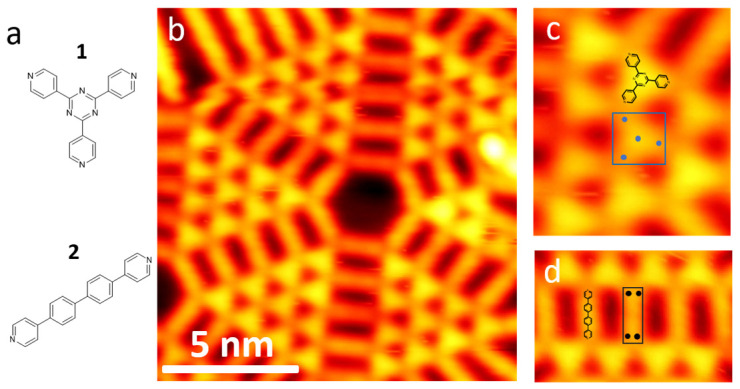
(**a**) Chemical structures of molecules **1** and **2**. (**b**) STM image of surface nanostructures formed by **1** and **2**. Zoomed-in area highlighting (**c**) molecule **1** and (**d**) molecule **2**. The manual annotations of bounding boxes and keypoints are overlapped on the corresponding molecules in (**c**,**d**). Scanning parameters: V_t_ = −1.0 V, I_t_ = 60 pA.

As a prototypical example, we selected a self-assembled molecular nanostructure composed of two molecules, one with a triangular and the other with a rectangular symmetry, the chemical structures of which are shown in Figure 2 [36]. For simplicity, we refer to these two molecules as molecule **1** (2,4,6-tri(Pyridin-4-yl)-1,3,5-triazine) and molecule **2** (4,4′-Di(Pyridin-4-Yl)-1,1′-Biphenyl). Figure 2b shows a typical STM image of the nanostructure where we can recognize the two molecules by their distinct shapes. Both of the molecules can be represented by four keypoints as marked by the blue dots in Figure 2c,d, where the blue dots mark the three-fold symmetry of **1** and the two-fold symmetry of **2**. In addition, a bounding box is defined to label the molecule. The complete dataset was generated by maintaining the labels of the molecules and applying different combinations of the augmentation techniques. Overall, we annotated only a dozen of molecules manually. 

mAP (mean Average Precision) is commonly used to assess the performance of the object detection algorithms. mAP@0.5 represents the average precision when the overlap between the predicted bounding boxes and ground truth boxes (usually measured by Intersection over Union, IOU) reaches 0.5:

$$\mathrm{mAP}@0.5=\frac{1}{N}\sum_{i=1}^{N}{\mathrm{AP}}_{0.5}^{\left(i\right)}$$

where *N* represents the total number of classes and ${\mathrm{AP}}_{0.5}^{\left(i\right)}$ denotes the average precision for class *i* at an IOU threshold of 0.5. In object detection tasks, an overlap of 0.5 is considered as a standard threshold, and mAP@0.5 evaluates the performance for most practical applications. In addition, mAP@0.5:0.95 represents the average precision when the overlap ranges from 0.5 to 0.95, that is,

$$\mathrm{mAP}@0.5:0.95=\frac{1}{N}\sum_{i=1}^{N}{\mathrm{AP}}_{0.5:0.95}^{\left(i\right)}$$

where *N* represents the total number of classes and ${\mathrm{AP}}_{0.5:0.95}^{\left(i\right)}$ represents the average precision for class *i* with the IOU threshold ranging from 0.5 to 0.95. This metric provides a more comprehensive evaluation, as it considers the precision variation under different overlap requirements. In this case, the mAP@0.5:0.95 provides a more complete assessment, as it considers precision changes at different overlap levels. 

To achieve better model performance, we applied and tested different data augmentation techniques and their combinations [37,38]. First, we selected a representative subset of molecules from the original dataset and rotated each molecule by 15 degrees until a full rotation is completed. Then, we performed vertical, horizontal, and diagonal flips on each image. This approach allowed us to capture different orientations of the molecules, thus enriching the dataset. We refer to the data augmentation of image rotation and flipping as S1. Next, we applied image blurring, dropout, and elastic transformations as S2, and operations of image hue, saturation, and brightness as S3 (see more details of the augmentation in Table S2). 

**Figure 3:**
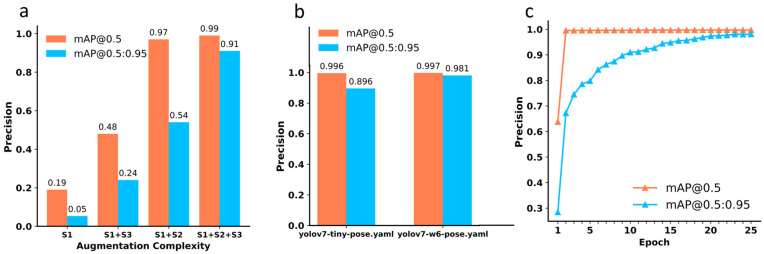
(**a**) The ML model performance with different data augmentation methods. (**b**) Model performance with different configuration files of the YOLO model. (**c**) The training processes of the ML model at mAP@0.5 (denoted by the coral line) and mAP@0.5:0.95 (denoted by the cyan line).

We then created different datasets by combining these three data augmentation levels and trained the ML models accordingly. The results obtained are shown in Figure 3, from which we realize that that using only S1 for data augmentation results in the mAP@0.5 and mAP@0.5:0.95 scores of 19.8% and 5.2%, respectively. However, when taking S1 and S3 for data augmentation, the mAP@0.5 and mAP@0.5:0.95 scores increase to 48% and 24.2%, respectively. Remarkably, the combination of S1 and S2 results in a significant improvement in the mAP@0.5, but the mAP@0.5:0.95 score is not dramatic. However, when all the three data augmentation levels are applied, we achieve excellent model performance with mAP@0.5 and mAP@0.5:0.95 scores of 99.6% and 91.1%, respectively.

We also tested different configuration files for training under the same dataset to make the entire model more compatible with our work. The yolov7-tiny-pose.yaml and yolov7-w6-pose.yaml are two different configuration files, and their difference lies in the depth and width of the model. The file yolov7-tiny-pose.yaml is a relatively small model that is suitable for use when computing resources are limited, while yolov7-w6-pose.yaml is a larger model that is suitable for scenarios that require higher accuracy. As displayed in Figure 3b, the configuration file of yolov7-tiny-pose.yaml does not perform as well as yolov7-w6-pose.yaml. Therefore, we used the yolov7-w6-pose.yaml configuration file to build the ML model. Eventually, our model achieves a maximum mAP@0.5 of 99.7% and mAP@0.5:0.95 of 99.1%, reflecting a high performance for the molecular imaging recognition (Figure 3c).

**Figure 4:**
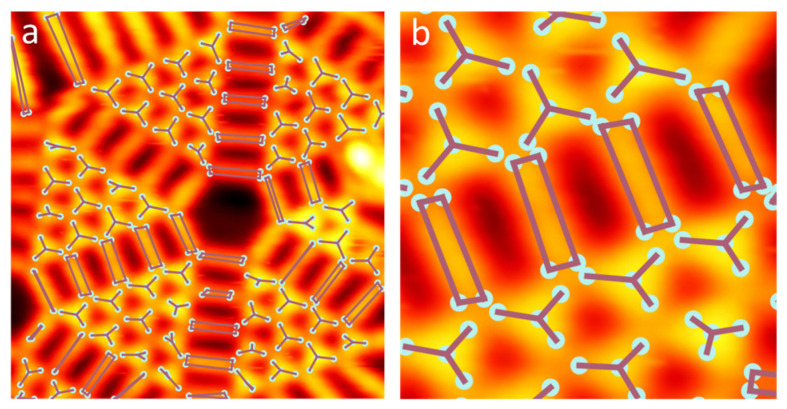
(**a**) The output of our machine learning model in which the molecules are labeled with keypoints. The keypoints are connected to their nearest neighbors with edges. (**b**) A close-up look at the output image., where we could recognize the triangular shape of **1** and the rectangular shape of **2**.

In addition to achieving the object detection tasks, our model was used to output keypoints of objects [39]. As demonstrated in Figure 4, the keypoints are predicted for each molecule, from which the triangle-shaped molecule **1** and the rectangle-shaped molecule **2** can be easily recognized through drawing the connection between the keypoints to their nearest neighbors. Notably, the entire deep learning process is run directly on a personal computer, and only requires 2–3 h for model training. Furthermore, predicting the STM image and outputting the results takes only a fraction of a second (see more recognition cases in Supplementary Figures S2–S4).

**Figure 5:**
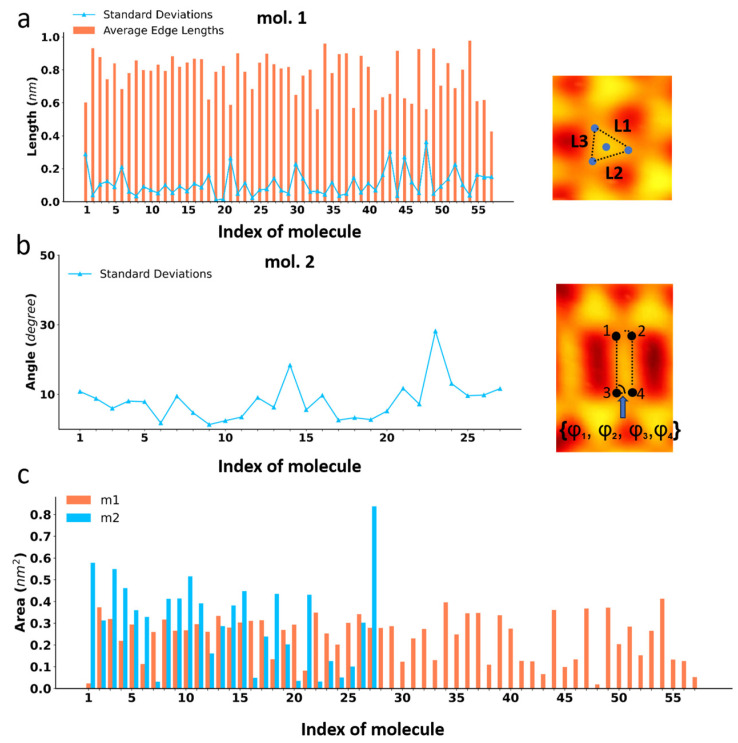
(**a**) Statistics on the average side length and its standard deviation of molecule **1**, reflecting its three-fold symmetry. On the right, we show how the side lengths of the molecule are obtained. (**b**) Statistics on the standard deviation of the internal angles of molecule **2** with respect to the theoretical orthogonal angles, reflecting the two-fold symmetry of **2**. On the right, we show how the internal angles are obtained. (**c**) Statistics on the areas of the two molecules which correspond to the areas enclosed by the keypoints.

With the predicted keypoints, we can obtain a wealth of information regarding the investigated molecules. For example, we can analyze the symmetry of the molecules. From our prior knowledge, we know that molecule **1** has a three-fold symmetry while molecule **2** is two-fold symmetric. For **1**, the connected edges of the predicted keypoints form triangles, whose side lengths can be determined as well as the average and the computed standard deviation. The smaller the standard deviation, the more it indicates that the molecule possesses three-fold symmetry. The results are shown in Figure 5a. For **2**, the connected edges using the predicted keypoints establish quadrilaterals, from which the degrees of each interior angle are calculated and their standard deviations from the theoretical orthogonal angles are determined. Again, the smaller the standard deviation, the more it indicates that the molecules have a rectangular shape (shown in Figure 5b). In addition, with the predicted keypoints, we can also determine the actual area of each molecule. Based on the scale in the STM image, the real size of each pixel in the image can be obtained, allowing the calculation of the actual size of the molecules by multiplying it with the occupied pixels surrounded by the keypoints. The results are shown in Figure 5c.

**Figure 6:**
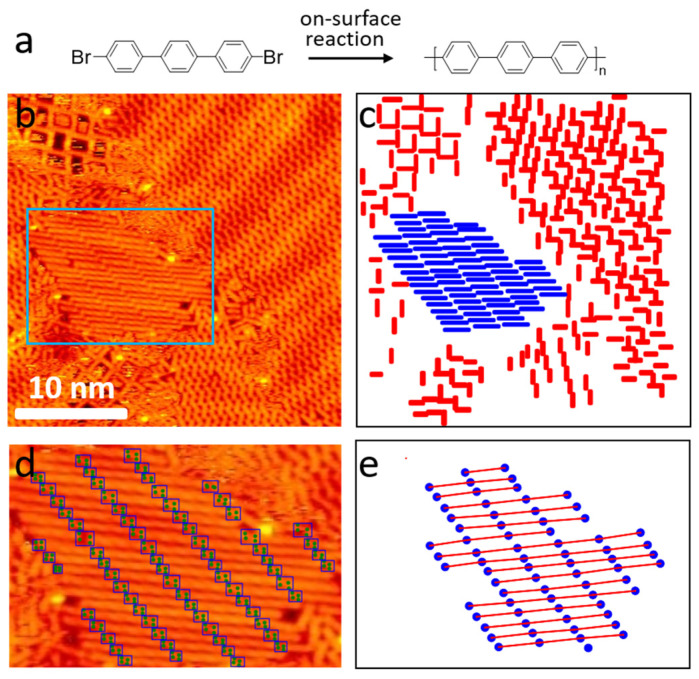
(**a**) The reaction scheme of the on-surface polymerization of the dibromo-terphenyl molecule. (**b**) STM image after the reaction. (**c**) The output annotations generated by the ML model. Red represents the unit molecules and blue represents the polymerized molecules after the reaction. (**d**) STM image with the endpoint detection of the polymerized molecules for the region indicated in (**b**). (**e**) The predicted endpoints are connected after calculating the distances between them and finding those with lengths that are integral multiples of the length of a single molecule. The endpoints are represented by blue dots.

Lastly, we showcase the usefulness of our deep learning framework by analyzing the surface nanostructures of on-surface synthesis [16]. Figure 6a shows a schematic representation of a well-studied polymerized reaction of a bromine-functionalized polycyclic aromatic hydrocarbon on the surface of Au(111), which generates a polyphenylene chain. In Figure 6b, we show a typical STM image after the reaction. This image can be analyzed using two algorithms.

First, since the sample contains molecules before and after the reaction as reflected by their different lengths, we annotated both the reactants and product molecules. After training the model and outputting the predicted labels, we can determine the reaction yield from the image. As indicated in Figure 6c, the red lines suggest molecules without C-C coupling, and the blue lines suggest the polymerized molecules. We can thus calculate the reaction yield simply by dividing the total sizes of the blue and red lines by the size of the blue ones.

The second method involves determining the length of the polymerized chain by annotating the endpoints of the molecules as indicated in Figure 6d. The polymers after the on-surface reaction are commonly not of uniform length, which can be dimers, trimers, or even longer. Therefore, annotating a large number of oligomers is impractical. We annotated the characteristic gaps between the oligomers, allowing our model to predict the endpoints of the polymerized chain. This allows us to determine the number of molecules in a polymer simply by dividing the length of the chain by the length of a single molecule. The potential of this deep learning framework lies in the prediction and identification of reaction products in on-surface synthesis, particularly in complex systems with different molecules. With the detection of the keypoints of target molecules, we can quickly and accurately determine the composition and structure of reaction products, providing valuable information for further analyses. 

## 3. Conclusions

In summary, we developed a deep learning framework based on the YOLOv7 algorithm for the automated analysis of surface nanostructures in STM images. Our computer vision framework introduces keypoints into STM image recognition. By carefully selecting image augmentation techniques, we only need to include limited STM images containing a dozen of molecules for training. In the testing case of a standard STM image, our model achieves an excellent performance of an mAP@0.5 of 99.52% and an mAP@0.5:0.95 of 99.93%. We demonstrate that this framework not only recognizes different types of molecules but also provides keypoints for each molecule, which can be used for further data analysis. Moreover, this model is lightweight, easy to operate, and highly accurate, making it a potential general solution for the automated detection and analysis of molecular STM images.

## Data Availability

The main data supporting the findings of this study are available within the paper and the supporting information. Publicly available codes can be found at: https://github.com/gggg0034/yolov7_keypoint (accessed on 10 July 2023).

## Supplementary Materials

The following supporting information can be downloaded at: https://www.mdpi.com/article/10.3390/molecules28145387/s1, Figure S1: The Backbone and Head of yolov7; Figure S2: Identification of single-component molecules; Figure S3: Identification of dual-component molecules; Figure S4: Identification of two highly similar molecules; Table S1: Effects and descriptions of different image augmentation techniques; Table S2: Different combinations of data augmentation. Table S3: Comparison of other deep-learning models. References [40,41] are cited in Supplementary Materials.

## References

[1] Di Giovannantonio M., Fasel R. (2022). On-Surface Synthesis and Atomic Scale Characterization of Unprotected Indenofluorene Polymers. J. Polym. Sci..

[2] Wang J., Niu K., Xu C., Zhu H., Ding H., Han D., Zheng Y., Xi J., You S., Deng C. (2022). Influence of Molecular Configurations on the Desulfonylation Reactions on Metal Surfaces. J. Am. Chem. Soc..

[3] Kang F., Sun L., Gao W., Sun Q., Xu W. (2023). On-Surface Synthesis of a Carbon Nanoribbon Composed of 4–5–6–8-Membered Rings. ACS Nano.

[4] Kinikar A., Di Giovannantonio M., Urgel J.I., Eimre K., Qiu Z., Gu Y., Jin E., Narita A., Wang X.-Y., Müllen K. (2022). On-Surface Polyarylene Synthesis by Cycloaromatization of Isopropyl Substituents. Nat. Synth..

[5] Liu L., Klaasen H., Witteler M.C., Schulze Lammers B., Timmer A., Kong H., Moenig H., Gao H.Y., Neugebauer J., Fuchs H. (2021). Polymerization of Silanes through Dehydrogenative Si–Si Bond Formation on Metal Surfaces. Nat. Chem..

[6] Mallada B., de la Torre B., Mendieta-Moreno J.I., Nachtigallová D., Matěj A., Matoušek M., Mutombo P., Brabec J., Veis L., Cadart T. (2021). On-Surface Strain-Driven Synthesis of Nonalternant Non-Benzenoid Aromatic Compounds Containing Four- to Eight-Membered Rings. J. Am. Chem. Soc..

[7] Sun Q., Yan Y., Yao X., Müllen K., Narita A., Fasel R., Ruffieux P. (2021). Evolution of the Topological Energy Band in Graphene Nanoribbons. J. Phys. Chem. Lett..

[8] Zhu X., Liu Y., Pu W., Liu F.-Z., Xue Z., Sun Z., Yan K., Yu P. (2022). On-Surface Synthesis of C144 Hexagonal Coronoid with Zigzag Edges. ACS Nano.

[9] Jung T.A., Schlittler R.R., Gimzewski J.K. (1997). Conformational Identification of Individual Adsorbed Molecules with the STM. Nature.

[10] Wyrick J., Wang X., Namboodiri P., Kashid R.V., Fei F., Fox J., Silver R. (2022). Enhanced Atomic Precision Fabrication by Adsorption of Phosphine into Engineered Dangling Bonds on H–Si Using STM and DFT. ACS Nano.

[11] Wang L., Xia Y., Ho W. (2022). Atomic-Scale Quantum Sensing Based on the Ultrafast Coherence of an H2 Molecule in an STM Cavity. Science.

[12] Meng T., Lu Y., Lei P., Li S., Deng K., Xiao X., Ogino K., Zeng Q. (2022). Self-Assembly of Triphenylamine Macrocycles and Co-Assembly with Guest Molecules at the Liquid–Solid Interface Studied by STM: Influence of Different Side Chains on Host–Guest Interaction. Langmuir.

[13] Moreno D., Parreiras S.O., Urgel J.I., Muñiz-Cano B., Martín-Fuentes C., Lauwaet K., Valvidares M., Valbuena M.A., Gallego J.M., Martínez J.I. (2022). Engineering Periodic Dinuclear Lanthanide-Directed Networks Featuring Tunable Energy Level Alignment and Magnetic Anisotropy by Metal Exchange. Small.

[14] Lyu C.-K., Gao Y.-F., Gao Z.-A., Mo S.-Y., Hua M.-Q., Li E., Fu S.-Q., Chen J.-Y., Liu P.-N., Huang L. (2022). Synthesis of Single-Layer Two-Dimensional Metal–Organic Frameworks M_3_(HAT)_2_ (M = Ni, Fe, Co, HAT = 1,4,5,8,9,12-Hexaazatriphenylene) Using an On-Surface Reaction. Angew. Chem..

[15] Liu J., Li J., Xu Z., Zhou X., Xue Q., Wu T., Zhong M., Li R., Sun R., Shen Z. (2021). On-Surface Preparation of Coordinated Lanthanide-Transition-Metal Clusters. Nat. Commun..

[16] Jiang H., Lu J., Zheng F., Zhu Z., Yan Y., Sun Q. (2023). Steering On-Surface Polymerization through Coordination with a Bidentate Ligand. Chem. Commun..

[17] Zhu Z., Lu J., Zheng F., Chen C., Lv Y., Jiang H., Yan Y., Narita A., Müllen K., Wang X. (2022). A Deep-Learning Framework for the Automated Recognition of Molecules in Scanning-Probe-Microscopy Images. Angew. Chem. Int. Ed..

[18] Milošević D., Vodanović M., Galić I., Subašić M. (2022). Automated Estimation of Chronological Age from Panoramic Dental X-ray Images Using Deep Learning. Expert Syst. Appl..

[19] Zheng F., Zhu Z., Lu J., Yan Y., Jiang H., Sun Q. (2023). Predicting the HOMO-LUMO Gap of Benzenoid Polycyclic Hydrocarbons via Interpretable Machine Learning. Chem. Phys. Lett..

[20] Krull A., Hirsch P., Rother C., Schiffrin A., Krull C. (2020). Artificial-Intelligence-Driven Scanning Probe Microscopy. Commun. Phys..

[21] Alldritt B., Hapala P., Oinonen N., Urtev F., Krejci O., Federici Canova F., Kannala J., Schulz F., Liljeroth P., Foster A.S. (2020). Automated Structure Discovery in Atomic Force Microscopy. Sci. Adv..

[22] Hellerstedt J., Cahlík A., Švec M., Stetsovych O., Hennen T. (2022). Counting Molecules: Python Based Scheme for Automated Enumeration and Categorization of Molecules in Scanning Tunneling Microscopy Images. Softw. Impacts.

[23] Li J., Telychko M., Yin J., Zhu Y., Li G., Song S., Yang H., Li J., Wu J., Lu J. (2021). Machine Vision Automated Chiral Molecule Detection and Classification in Molecular Imaging. J. Am. Chem. Soc..

[24] Gordon O.M., Hodgkinson J.E., Farley S.M., Hunsicker E.L., Moriarty P.J. (2022). Automated Searching and Identification of Self-Organized Nanostructures. Nano Lett..

[25] Yan Y., Zheng F., Qie B., Lu J., Jiang H., Zhu Z., Sun Q. (2023). Triangle Counting Rule: An Approach to Forecast the Magnetic Properties of Benzenoid Polycyclic Hydrocarbons. J. Phys. Chem. Lett..

[26] Kang J., Yoo Y.J., Park J.-H., Ko J.H., Kim S., Stanciu S.G., Stenmark H.A., Lee J., Mahmud A.A., Jeon H.-G. Deepgt: Deep Learning-Based Quantification of Nanosized Bioparticles in Bright-Field Micrographs of Gires-Tournois Biosensor. NANOTODAY-D-23-00370. https://ssrn.com/abstract=4428599.

[27] Faraz K., Grenier T., Ducottet C., Epicier T. (2022). Deep Learning Detection of Nanoparticles and Multiple Object Tracking of Their Dynamic Evolution during in Situ ETEM Studies. Sci. Rep..

[28] Newby J.M., Schaefer A.M., Lee P.T., Forest M.G., Lai S.K. (2018). Convolutional Neural Networks Automate Detection for Tracking of Submicron-Scale Particles in 2D and 3D. Proc. Natl. Acad. Sci. USA.

[29] Nartova A.V., Mashukov M.Y., Astakhov R.R., Kudinov V.Y., Matveev A.V., Okunev A.G. (2022). Particle Recognition on Transmission Electron Microscopy Images Using Computer Vision and Deep Learning for Catalytic Applications. Catalysts.

[30] Choudhary K., DeCost B., Chen C., Jain A., Tavazza F., Cohn R., Park C.W., Choudhary A., Agrawal A., Billinge S.J. (2022). Recent Advances and Applications of Deep Learning Methods in Materials Science. Npj Comput. Mater..

[31] Wang C.-Y., Bochkovskiy A., Liao H.-Y.M. YOLOv7: Trainable Bag-of-Freebies Sets New State-of-the-Art for Real-Time Object Detectors. Proceedings of the IEEE/CVF Conference on Computer Vision and Pattern Recognition.

[32] Okunev A.G., Nartova A.V., Matveev A.V. Recognition of Nanoparticles on Scanning Probe Microscopy Images Using Computer Vision and Deep Machine Learning. Proceedings of the 2019 International Multi-Conference on Engineering, Computer and Information Sciences (SIBIRCON).

[33] Ullah M.B. CPU Based YOLO: A Real Time Object Detection Algorithm. Proceedings of the 2020 IEEE Region 10 Symposium (TENSYMP).

[34] Feng H., Mu G., Zhong S., Zhang P., Yuan T. (2022). Benchmark Analysis of YOLO Performance on Edge Intelligence Devices. Cryptography.

[35] Zhou S., Cai K., Feng Y., Tang X., Pang H., He J., Shi X. (2023). An Accurate Detection Model of Takifugu Rubripes Using an Improved YOLO-V7 Network. J. Mar. Sci. Eng..

[36] Lu J., Jiang H., Yan Y., Zhu Z., Zheng F., Sun Q. (2022). High-Throughput Preparation of Supramolecular Nanostructures on Metal Surfaces. ACS Nano.

[37] Ding K., Xu Z., Tong H., Liu H. (2022). Data Augmentation for Deep Graph Learning: A Survey. SIGKDD Explor. Newsl..

[38] Perez L., Wang J. (2017). The Effectiveness of Data Augmentation in Image Classification Using Deep Learning. arXiv.

[39] Maji D., Nagori S., Mathew M., Poddar D. YOLO-Pose: Enhancing YOLO for Multi Person Pose Estimation Using Object Keypoint Similarity Loss. Proceedings of the IEEE/CVF Conference on Computer Vision and Pattern Recognition.

[40] Le V.-H. Automatic 3D Hand Pose Estimation Based on YOLOv7 and HandFoldingNet from Egocentric Videos. Proceedings of the 2022 RIVF International Conference on Computing and Communication Technologies (RIVF).

[41] Roth K., Pemula L., Zepeda J., Schölkopf B., Brox T., Gehler P. (2022). Towards Total Recall in Industrial Anomaly Detection. arXiv.

