# Supplementary material for: Applying a Deep-Learning-Based Keypoint Detection in Analyzing Surface Nanostructures
Source: Molecules. 2023 Jul 13;28(14):5387. doi: 10.3390/molecules28145387 (PMC10384857; doi:10.3390/molecules28145387)
Supplement: Supplementary file 1 [file molecules-28-05387-s001.zip › molecules-2480723-supplementary.pdf]

Supporting Information for

Applying a deep-learning-based keypoint detection in analyzing surface nanostructures

Table of Contents

1. YOLOv7 model structure

2. Image augmentation techniques

3. More recognition cases

4. Precision, recall, and mAP

5. Comparison with other ML models

6. User guideline

7. Methods

8. References

1. YOLOv7 model structure

a) YOLOv7 Overall Structure

First, the input image is resized to 640×640 [1] and it is input into the backbone network. Then, three layers of feature maps of different sizes are output through the head layer network. The predictive results are outputted through Rep and conv. Here, taking the COCO dataset as an example, 80 categories are output, and then each output (x, y, w, h, and o) is the coordinate position and background; 3 refers to the number of anchors, so each layer output is (80+5)×3 = 255 times the size of the feature map, which is the final output.

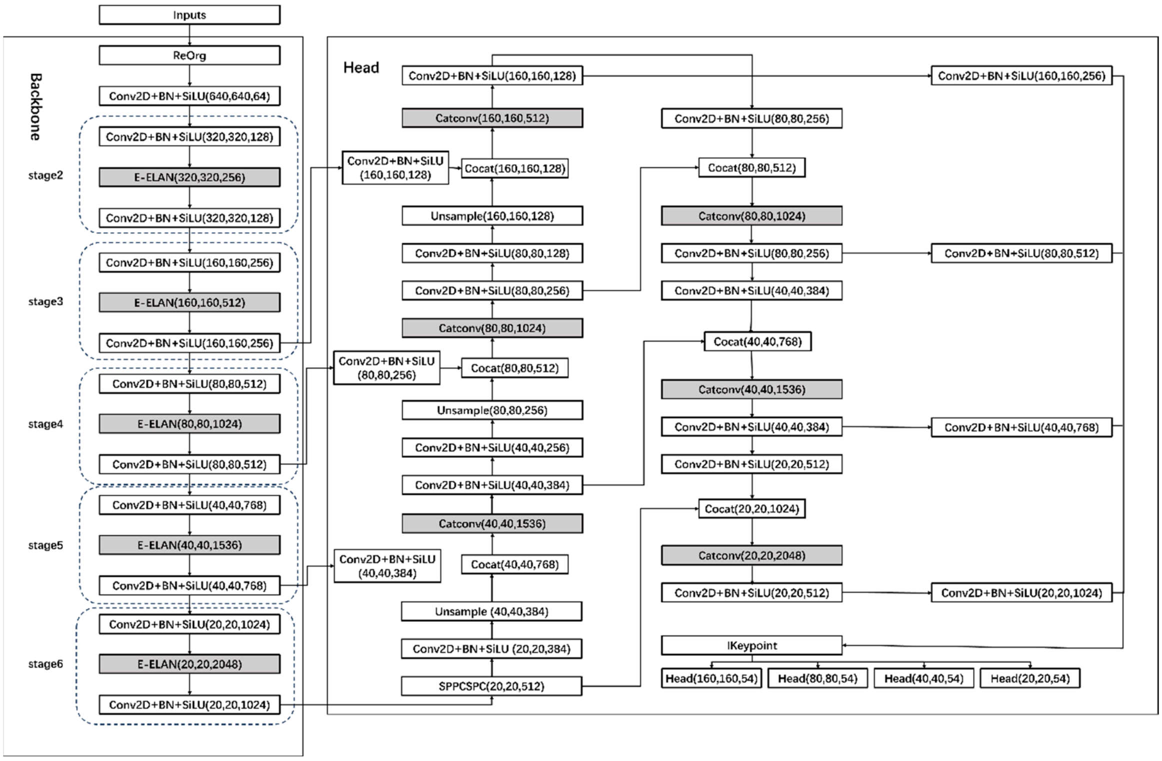

Figure S1. The Backbone and Head of YOLOv7.

b) Backbone

There are a total of 50 layers, first passing through 4 convolutional layers, mainly consisting of Conv + BN + SiLU [1] (hereinafter referred to as CBS). After four CBSs, the feature map changes to 160 * 160 * 128 size. Then, it goes through the ELAN module. ELAN is composed of multiple CBSs, and the sizes of the input and output features remain unchanged. The number of channels will change in the first two CBSs, and the input channels are consistent with the output channels in the subsequent ones.

After the last CBS, the output is the required channel. Overall, the backbone passes through four CBSs, then connects to an ELAN, and then there are three MP + ELAN outputs corresponding to the outputs of C3/C4/C5 with sizes of 80 * 80 * 512, 40 * 40 * 1024, and 20 * 20 * 1024, respectively. Each MP has 5 layers, and ELAN has 8 layers, so the total number of backbone layers is 4 + 8 + 13 * 3 = 51 layers. Starting from 0, the last layer is the 50th layer.

c) Head

YOLOv7 Head is actually a "pafpn" structure, the same as in the previous YOLOv4 and YOLOv5. First, for the 32-fold down-sampling feature map C5 outputted by the backbone, it goes through SPPCSP, and the number of channels changes from 1024 to 512. First, it fuses with C4 and C3 in a top-down manner to obtain P3, P4, and P5; then, it fuses with P4 and P5 in a bottom-up manner. This is basically the same as YOLOv5. The difference is that the CSP module in YOLOv5 has been replaced with the ELAN-H module, and the down-sampling has become the MP2 layer. For the P3, P4, and P5 outputs by "pafpn", the number of channels is adjusted through RepConv, and finally the 1×1 convolution is used to predict the class, keypoint [2], and bounding box.

2. Image augmentation techniques

We used the imgaug [3] library for data augmentation. While some data augmentation methods may not be obvious to humans, they render a completely new image for the computer, which is extremely useful for machine learning. In Table S1, we demonstrate the changes brought about by each different data augmentation method to the images.

Table S1. Effects and descriptions of different image augmentation techniques.

**Augmentation techniques**	**Image example**	**Description**
None	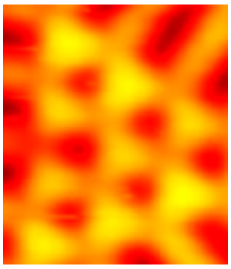	The original image

CoarseDropout	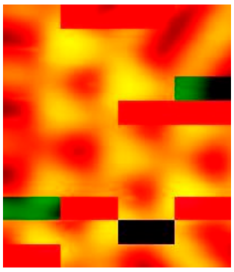	A coarser version of dropout that generates larger missing areas.
Elastic	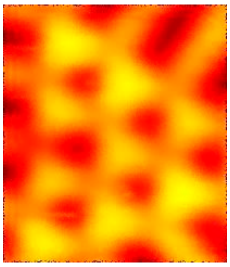	Transform images by moving pixels locally around using displacement fields.
Blurring	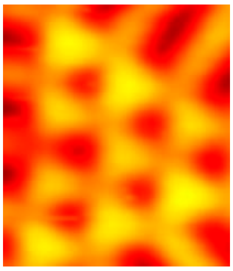	A Gaussian blur that simulates blur due to the camera being out of focus.
Brightness	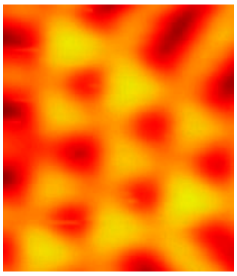	An operation that adjusts the brightness and will simulate various color conditions.
Hue	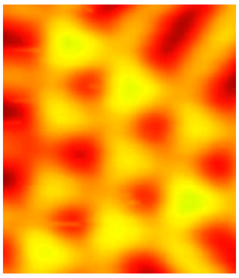	An operation that adds a value directly to the hue and will shift the color tones in the image.
Dropout	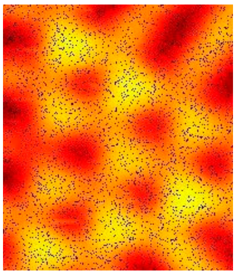	Set a certain fraction of pixels in images to zero.

Saturation	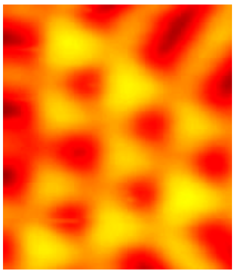	An operation that adds a value directly to the saturation and will change the intensity of colors in the image.
Rotation	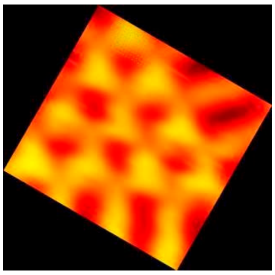	Rotate image.
Vertical flip	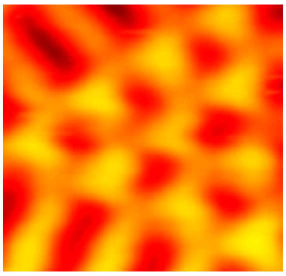	Flip the image upside down.
Horizontal flip	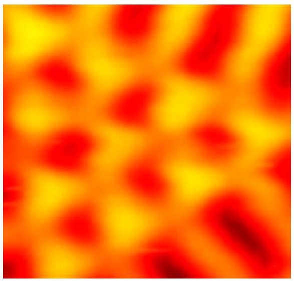	Flip the image left to right.
Diagonal flip	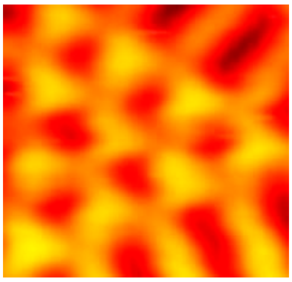	Flip the image diagonally.

Table S2. Different combinations of data augmentation.

	**Operation**
**S1**	Rotation, vertical, horizontal, and diagonal flips
**S2**	Blurring, dropout, and elastic
**S3**	Hue, saturation, and brightness

3. More recognition cases

In addition to the examples shown in the main text, we also conducted molecule identifications for other STM images. Under the same parameters, we achieved good performances of image recognitions and keypoint identifications. The following images show some examples. The first shows the identification of single-component molecules (Figure S2). Figure S3 shows the identification of dual-component molecules.

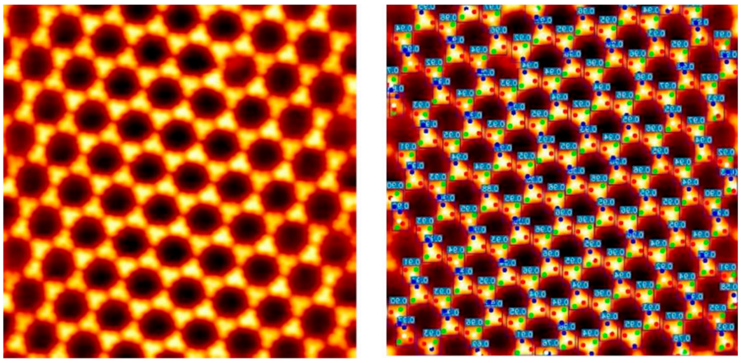

Figure S2. identification of single-component molecules.

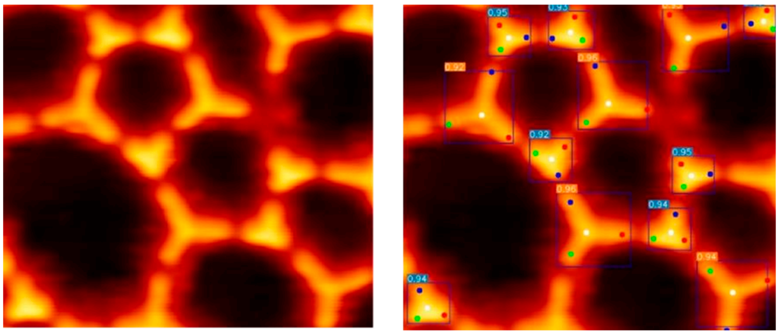

Figure S3. Identification of dual-component molecules.

Figure S4a shows the STM image of two highly similar molecules, **molecule 1** and **molecule 2**. Figure S4b represents the detection results obtained by our model, where the blue bounding box corresponds to **molecule 1** and the orange bounding box corresponds to **molecule 2**. Our model demonstrates an almost excellent detection accuracy, revealing its capability to identify molecules that even the human eye might struggle to differentiate due to their high similarity.

From these three additional examples, we are convinced of the accuracy of our model.

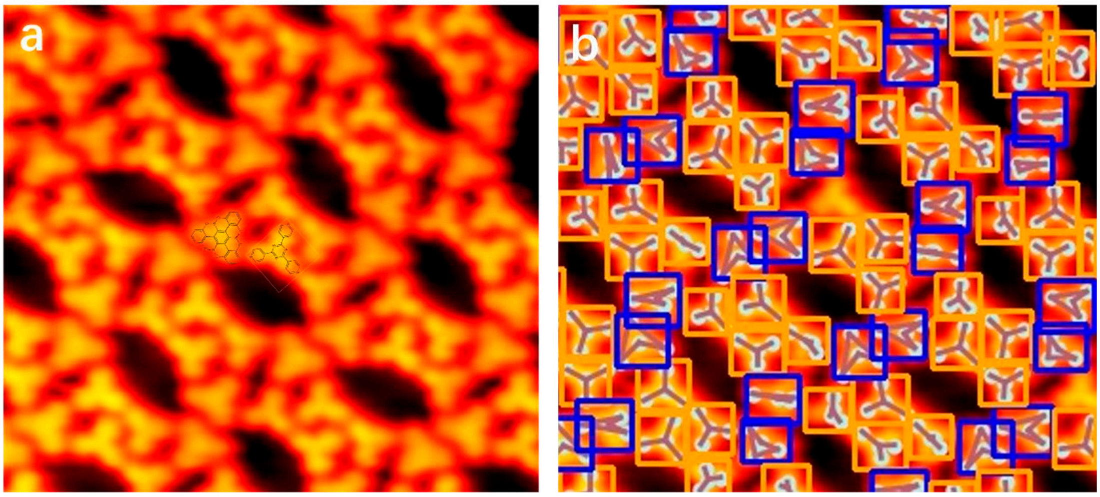

Figure S4. Identification of two highly similar molecules.

4. Precision, recall, and mAP

Precision, recall, and mean Average Precision (mAP) [4] are widely used performance metrics in the field of information retrieval and machine learning. Precision refers to the proportion of correctly identified positive results among the total returned results. It is also called positive predictive value. It is defined as

$$\text{Precision} = TP / (TP + FP)$$

where TP is the number of true positives and FP is the number of false positives. High precision indicates a low false-positive rate.

Recall, also known as sensitivity, hit rate, or true-positive rate, is the ratio of correctly identified positive results to the total actual positive cases. It is defined as

$$\text{Recall} = TP / (TP + FN)$$

where FN is the number of false negatives. High recall indicates that the model returns most of the relevant results.

Mean Average Precision (mAP) is a comprehensive metric used in information retrieval to measure the effectiveness of ranking algorithms in terms of both recall and precision. mAP calculates the average precision values at the point of each relevant document in the ranked order of retrieval. The formula involves calculating the Average Precision (AP) for each query and then taking the average over all queries:

$$\text{mAP} = (1 / Q) * \Sigma (\text{APq})$$

where Q is the total number of queries and APq is the Average Precision at each query, q. These metrics are critical for understanding the trade-off between precision and

recall, which is vital for evaluating models in many tasks, particularly in scenarios where false positives and false negatives have different costs.

5. Comparison with other ML models

Table S3. Comparison of other deep-learning models.

	**YOLO (You Only Look Once)**	**U-Net**	**Traditional CNN (Convolutional Neural Network)**
Task	Object detection	Image segmentation	Various computer vision tasks
Architecture	CNN-based with anchor boxes	CNN-based with encoder–decoder	CNN-based with various architectures
Network structure	Simple architecture using convolutional layers	Encoder extracts features and decoder maps features segmentation masks	Multiple layers (convolutional, pooling, and fully connected) for feature extraction
Data requirements	Labeled dataset with object bounding box annotations and class labels	Labeled dataset with pixel-level class labels	Labeled dataset suitable for the specific task
Advantages	Fast inference, capable of real-time object detection	Capable of pixel-level image segmentation, good performance in detailed segmentation tasks	Effective feature extraction, suitable for various computer vision tasks
Disadvantages	May have poor performance in detecting small objects and higher false-positive rates	Relatively slower compared to YOLO, requires more training data	Performance may vary depending on the specific task and architecture

From the above, it is clear that U-Net requires a larger dataset, while our STM images are obtained from our own laboratory, making it difficult to provide a large dataset.

Therefore, we are seeking a model that can work with a small amount of data. Additionally, we are interested in obtaining information regarding molecular keypoints, which is inaccessible through semantic segmentation models. Furthermore, considering future research projects, we require a model with excellent real-time detection capabilities to perform live scanning and data analysis with STM imaging. Considering all these factors, we chose the YOLO model.

6. User guideline

6.1 Programming environment requirements

Python 3.8 https://www.python.org/ We strongly recommend using anaconda to manage the python environment.

Pytorch 1.12.1 https://pytorch.org/ The main body of our YOLOv7 code comes from an open-source project. We also strongly recommend downloading the GPU version of pytorch.

Numpy 1.23.3 We suggest selecting a suitable version for numpy, otherwise the code would run incorrectly.

cudnn 8.1.10, cudatoolkit 11.3 If one wants to correctly use GPU resources and download the right version of Pytorch, one must determine the cudnn and cudnntoolkit versions and follow the guide document in which Pytorch gives the right version to choose.

Imgaug 0.4.0 https://github.com/aleju/imgaug-doc imgaug is a library for image augmentations in machine learning projects.

Note: In the above we only list the important libraries and corresponding precautions. The detailed program running environment is accessible publicly**:** https://github.com /gggg0034/yolov7_keypoint.

6.2 References of the hardware requirements

CPU 12th Gen intel(R) Core i9 – 12900H

GPU NVIDIA GeForce RTX 3070Ti Laptop GPU 8G

Memory 32G

1 epoch time cost： $\leq$ 8 minutes

Max batch-size: 5 images

Note: The above computer hardware configuration is just for reference. We recommend using a graphics card with at least 8G video memory.

6.3 Here we teach how to prepare a dataset and train it on this framework a) Select an image

Select an image that needs to be trained; we suggest cropping out some representative molecules as the original dataset, but one should notice that the image's bit depth must be 24 bit not 32 bit. If the bit depth of the image is 32 bit one can simply turn it into 24 bit by saving it as another jpg image.

b) Label training images

(1) open labelme

Open the Prompt console as an administrator, switch to the environment where labelme is installed, type labelme after the console and press enter.

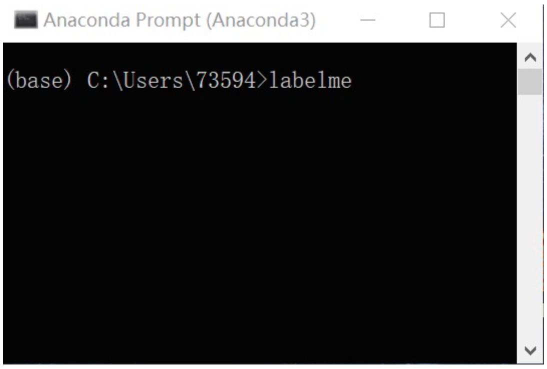

(2) Label molecules

Click on one of the buttons in the red box, find the picture needing to be annotated and open it.

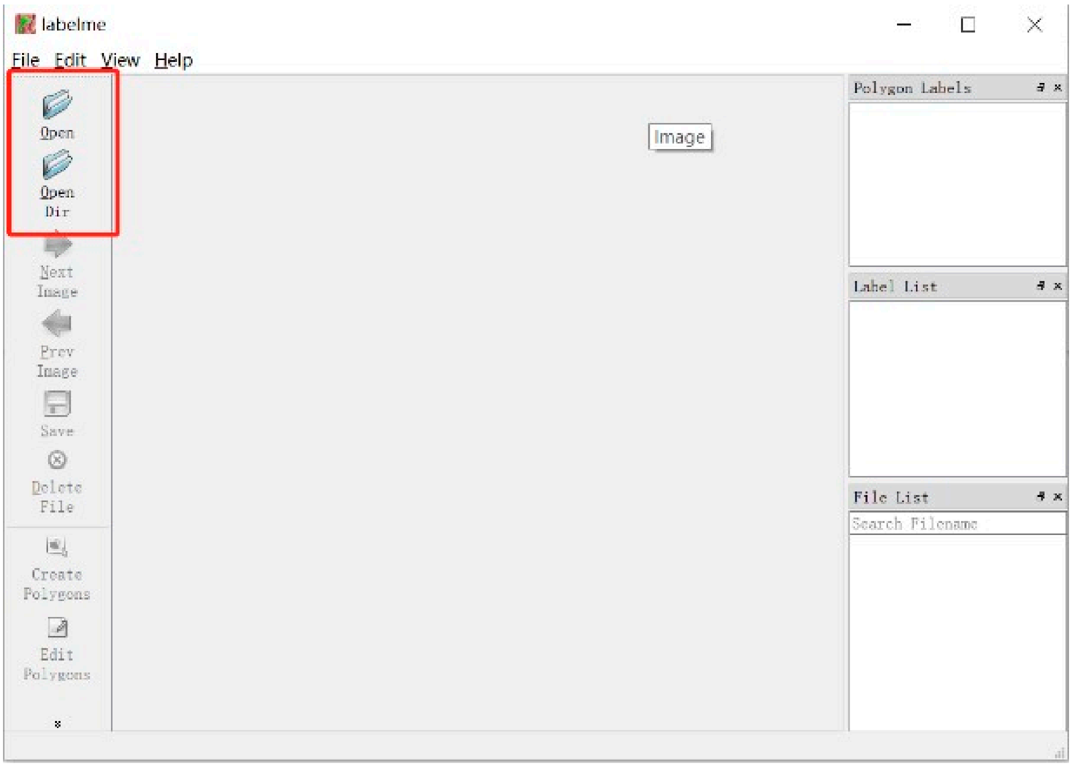

Due to the characteristics of the YOLO model, one can only annotate rectangles and points. If one wants to annotate more than one type of molecule, we recommend first annotating one type of molecule and then annotating the other types of molecules. Please indicate the number of annotations for each type of molecule. Then, click the save button, and one can receive a JSON annotation file with the same name as the image which it was imported in. **c) Augmentation**

After obtaining the original images and json files, place them in ./test/image&labels. First, modify the number of keypoints and the number of each type of molecules in the 'bbox_final.py', 'hrz_final.py', 'vet_final.py', and 'drop_final.py' files, and adjust the paths. Then, run 'workflow.py'. In this way, we can expand from a single dataset to approximately two thousand datasets.

Next, the json files need to be converted into xml files. The 'json2txt_feilei.py' file is used to output the txt format annotation files required by the YOLO model. Run 'workflow2.py' and 'workflow3.py' subsequently, which will copy the dataset to another folder, 'make-your-yolov5_dataset-main', to split the training and validation datasets. Simply running the 'voc_split_trainTestVal.py' and 'dataset_cg.py' scripts sequentially will do.

Finally, the generated dataset should be copied to the model folder, and it will then be ready for training.

d) Hyperparameters

The hyperparameter settings used in this study prior to training are outlined below:

- lr0: 0.01. Initial learning rate (SGD=1E-2, Adam=1E-3).
- lrf: 0.1. Final OneCycleLR learning rate (lr0 * lrf).
- momentum: 0.937. SGD momentum/Adam beta1.
- weight_decay: 0.0005. Optimizer weight decay (5e-4).
- warmup_epochs: 3.0. Number of warmup epochs (fractions allowed).
- warmup_momentum: 0.8. Initial momentum during warmup.
- warmup_bias_lr: 0.1. Initial bias learning rate during warmup.
- box: 0.05. Weight for the box loss.
- kpt: 0.005. Weight for the keypoint (kpt) loss.
- cls: 0.3. Weight for the classification (cls) loss.
- cls_pw: 1.0. Positive weight for the classification BCELoss.
- obj: 0.7. Weight for the objectness (obj) loss (scaled with pixels).
- obj_pw: 1.0. Positive weight for the objectness BCELoss.
- iou_t: 0.20. IoU training threshold.
- anchor_t: 4.0. Anchor-multiple threshold.
- anchors: 3. Number of anchors per output layer (0 to ignore).
- fl_gamma: 0.0. Focal loss gamma (efficientDet default gamma=1.5).
- hsv_h: 0.3. Image HSV-Hue augmentation (fraction).
- hsv_s: 0.3. Image HSV-Saturation augmentation (fraction).
- hsv_v: 0.3. Image HSV-Value augmentation (fraction).
- degrees: 0.0. Image rotation (+/- degrees).
- translate: 0.0. Image translation (+/- fraction).
- scale: 0.8. Image scale (+/- gain).
- shear: 0.5. Image shear (+/- degrees).
- perspective: 0.0. Image perspective (+/- fraction), range 0-0.001.
- flipud: 0.0. Image flip up-down (probability).
- fliplr: 0.0. Image flip left-right (probability).
- mosaic: 0.8. Image mosaic (probability).
- mixup: 0.3. Image mixup (probability).

e) Train

After opening the model folder, find the 'train_Ncla_nPoint.py' file.

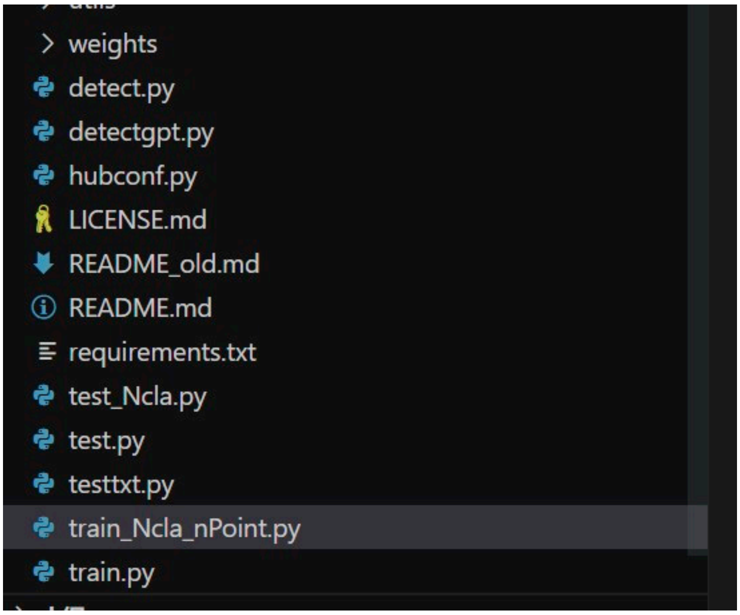

We suggest adjusting the model's parameters in the indicated area, primarily the 'epochs' and 'batch-size' parameters. 'Epochs' denotes how many rounds of the model to train, and 'batch-size' represents how many images the model can send to the GPU at once. For instance, with my computer's 8G memory, the highest setting is batch-size = 5, any higher will result in errors. Once these settings are adjusted, the model can begin training by running 'train_Ncla_nPoint.py'.

```python

if __name__ == '__main__':
    parser = argparse.ArgumentParser()
    parser.add_argument('--weights', type=str, default='weights/yolov7-w6-pose.pt', help='initial weights path')
    parser.add_argument('--cfg', type=str, default='cfg/yolov7-w6-pose.yaml', help='model.yaml path')
    parser.add_argument('--data', type=str, default='data/plate.yaml', help='data.yaml path')
    parser.add_argument('--hyp', type=str, default='data/hyp.pose.yaml', help='hyperparameters path')
    parser.add_argument('--epochs', type=int, default=50)
    parser.add_argument('--batch-size', type=int, default=5, help='total batch size for all GPUs')
    parser.add_argument('--img-size', nargs='+', type=int, default=[768, 768], help='[train, test] image sizes')
    parser.add_argument('--rect', action='store_true', help='rectangular training')
    parser.add_argument('--resume', nargs='?', const=True, default=False, help='resume most recent training')
    parser.add_argument('--nosave', action='store_true', help='only save final checkpoint')
    parser.add_argument('--notest', action='store_true', help='only test final epoch')
    parser.add_argument('--noautoanchor', action='store_true', help='disable autoanchor check')
    parser.add_argument('--evolve', action='store_true', help='evolve hyperparameters')
    parser.add_argument('--bucket', type=str, default='', help='gsutil bucket')
    parser.add_argument('--cache-images', action='store_true', help='cache images for faster training')
    parser.add_argument('--image-weights', action='store_true', help='use weighted image selection for training')
    parser.add_argument('--device', default='', help='cuda device, i.e. 0 or 0,1,2,3 or cpu')
    parser.add_argument('--multi-scale', action='store_true', help='vary img-size +/- 50%%')
```

f) Detect

After the training is completed, one needs to open the train folder in the run directory to find the latest training data. There is a weight folder within, and the 'best.pt' inside represents the best weight file among all the epochs trained, while 'last.pt'

represents the weight file outputted by the last epoch. Generally, we use 'best.pt' for prediction. Copy 'best.pt' to the weights folder in the main directory, open 'detect.py' in the compiler, and ensure that the 'weights' parameter defaults to the 'best.pt' just copied. This allows the model to run the 'detect.py' file for prediction. However, the images must be placed in the 'data\images' directory for prediction. Therefore, the image with labels will be in the runs/detect

file. The fifth parameter can indeed affect the detection/classification ranges, and

```
if __name__ == '__main__':
    parser = argparse.ArgumentParser()
    parser.add_argument('--weights', nargs='+', type=str, default='weights/best.pt', help='model.pt path(s)')
    parser.add_argument('--source', type=str, default='data/images', help='source')  # file/folder, 0 for webcam
    parser.add_argument('--img-size', nargs= '+', type=int, default=768, help='inference size (pixels)')
    parser.add_argument('--conf-thres', type=float, default=0.55, help='object confidence threshold')
    parser.add_argument('--iou-thres', type=float, default=0.25, help='IOU threshold for NMS')
```

this parameter can be adjusted to achieve the desired results.

7. Methods of

STM characterization.

We used a custom-designed commercial low-temperature STM system (Bosezi (Beijing) Co. Ltd.) for in situ characterization under ultra-high vacuum conditions of base pressures below 1×10^{-10} mbar. The single crystals (MaTeck GmbH) were cleaned by several cycles of argon sputtering and annealing under UHV conditions until large terraces separated by monatomic steps were achieved. The measurements were performed at liquid nitrogen temperature (~ 77.6 K) if not stated otherwise. STM imaging was performed with the constant-current mode at typical bias ranges of -1.0 to -2.0 V and current ranges of 50 to 150 pA.

Sample preparation.

The molecule precursors are commercially available. After degassing under UHV condition, the molecular precursors were thermally evaporated from a three-fold organic evaporator onto the metal surfaces in sequential order. The sublimation rates of both molecular precursors were monitored by a quartz crystal microbalance (SQM160, INFICON). We developed a LabVIEW based program to ensure that the molecular evaporation rate was stable for molecular evaporation.

Machine learning model and the program.

a. YOLOv7. The YOLO algorithm is a one-stage method that stands for You Only Look Once. It is a neural network that can output results by looking at an image only once. YOLO has released seven versions so far, with YOLOv1 laying the foundation for the entire YOLO series and subsequent YOLO algorithms constantly improving and innovating on it. The YOLO algorithm uses a single CNN model to achieve end-to-end object detection. The core idea is to use the entire image as the input of the network and directly regress the position of bounding boxes and their categories at the output layer.

b. *Image augmentation.* Data augmentation technology is used to obtain a large amount of image data. In the data augmentation process, we first performed geometric augmentations, followed by the other augmentation techniques. It is worth noting that the orientations and positions of molecules in the images after geometric augmentations have changed, which means that the labeling information in the corresponding image label files also needs to be changed accordingly to ensure the validity of the labeled data.

c. *Programming environment and hardware*. The program of the deep learning framework was run on a personal computer. The requirements of the programming environment and the references of the hardware can be found in Supplementary Section 6.

8. References

1. Wang, C.-Y.; Bochkovskiy, A.; Liao, H.-Y.M. YOLOv7: Trainable Bag-of-Freebies Sets New State-of-the-Art for Real-Time Object Detectors.; 2023; pp. 7464–7475.
2. Le, V.-H. Automatic 3D Hand Pose Estimation Based on YOLOv7 and HandFoldingNet from Egocentric Videos. In Proceedings of the 2022 RIVF International Conference on Computing and Communication Technologies (RIVF); December 2022; pp. 161–166.
3. Ding, K.; Xu, Z.; Tong, H.; Liu, H. Data Augmentation for Deep Graph Learning: A Survey. *ACM SIGKDD Explor. Newsl.* **2022**, *24*, 61–77.
4. Roth, K.; Pemula, L.; Zepeda, J.; Schölkopf, B.; Brox, T.; Gehler, P. Towards Total Recall in Industrial Anomaly Detection.; 2022; pp. 14318–14328.
